# SnO_2_@ZnO nanocomposites doped polyaniline polymer for high performance of HTM-free perovskite solar cells and carbon-based

**DOI:** 10.1038/s41598-022-24829-8

**Published:** 2022-12-07

**Authors:** Faezeh Arjmand, Zahra Golshani, Shahab Maghsoudi, Atena Naeimi, S. Jamiladin Fatemi

**Affiliations:** 1 Department of Chemistry, Shahid Bahonar University of Kerman, Kerman, 76169-133 Iran; 2 Department of Chemistry, Faculty of Science, University of Jiroft, Jiroft, 7867161167 Iran

**Keywords:** Chemistry, Nanoscience and technology

## Abstract

Herein, at first, green SnO_2_@ZnO nanocomposites were synthesized using *Calotropis* plant extract as an electron transfer material (ETM) to fabricate low-temperature-processed perovskite solar cells (PSCs). Then, the polyaniline (PANI) polymer was applied as an efficient additive to improve perovskite film quality. Under the effects of the small content of PANI additive, the quality of perovskite films is enhanced, which showed higher crystallinity in (110) crystal plane; also, the perovskite grains were found to be enlarged from 342 to 588 nm. The power conversion efficiency (PCE) of the prepared PSCs with SnO_2_@ZnO.PANI nanocomposites electron transfer layer (ETL) increased by 3.12%, compared with the PCE of SnO_2_@ZnO nanocomposites. The perovskite devices using SnO_2_@ZnO.PANI nanocomposites ETL have shown good stability during 480 h of tests. Furthermore, the optimal PSCs were fabricated by the mp-TiO_2_/SnO_2_@ZnO.PANI nanocomposites as ETL, which has a power conversion efficiency of 15.45%. We expect that these results will boost the development of low-temperature ETL, which is essential for the commercializing of high-performance, stable, and flexible perovskite solar cells.

## Introduction

Organic–inorganic perovskite materials (such as CH_3_NH_3_PbI_3_ = MAPbI_3_) have been successfully used in solar cells applications because of their wide absorption, high light absorption coefficient in the visible region, high ambipolar conductivity, long hole/electron diffusion length, suitable and tunable band gap, and excellent carrier transportation^1,2^. However, several studies have shown that the performance of perovskite solar cells (PSCs) depends on factors beyond the active layer, such as electron transfer layer (ETL) optimization^3^. The electron transfer layer plays a vital role in high performance PSCs, which increases the directional charge transfer of perovskite materials, and reduces the recombinant charge^4^. Generally, TiO_2_ is by far the most commonly adopted as the ETL of PSCs, which can effectively extract the electrons, block the holes and has also been used as a scaffold for the perovskite layer^5,6^. To crystallize the TiO_2_ layer, and increase electrical conductivity, TiO_2_ coating require a high temperature (> 450 °C) sintering step. Compared to other ETLs (such as ZnO), TiO_2_ has lower electron mobility. It also has numerous surfaces, and bulk flaws that impair the performance, and stability of photovoltaic devices. The UV instability of TiO_2_ in the face of ultraviolet light rapidly reduces the performance of PSCs by degrading organic components in the PSCs^6,7^. Low-temperature ETMs have received much attention from the (photovoltaic) PV community in recent years as a solution to this problem^8^. The various metal oxides that sinter at low temperatures, such as ZnO, SnO_2_, In_2_O_3_, and WO_3_ are considered alternative electron transfer materials for TiO_2_^9–12^. SnO_2_, one of these metal oxides, is an effective electron transfer layer in perovskite solar cells because it has a suitable energy level for perovskite electron injection. Additionally, SnO_2_-based PSCs have outstanding environmental resilience^13^. On electronic extraction, the ZnO displays high electronic mobility, but substantial surface recombination between the electron and hole can occur due to a surface defect that is inherent to the material. However, the low-temperature produced ZnO-based PSCs are unstable in the environment. When, the ZnO directly contacts with the perovskite crystals, the thermal decomposition of the perovskite film is driven by acid–base chemistry at the ZnO/CH_3_NH_3_PbI_3_ interface; deprotonation of the methylammonium cation by the ZnO surface leads to the formation of methylamine and PbI_2_^14^. Thus, the SnO_2_@ZnO nanocomposite, which combines the benefits of SnO_2_, and ZnO, is the optimal electron transport layer, in which the SnO_2_ nanoparticles give a stable energy level alignment for electron injection, and the ZnO nanoparticles provide high electron mobility. However, the SnO_2_ nanoparticles, due to van der Waals interactions between nanoparticles, tend to form large agglomerates spontaneously^15^. The interface between the electron transfer layer and the perovskite usually requires a modification process to increase the performance of the PSC^16^. The well-developed perovskite films include inactivated grain boundaries (GBs), large-sized crystal growth, high crystallinity, and low density of trapped defect. The causes of perovskite film defects include moisture, temperature, ultraviolet-A rays (UV-A), and bias voltage. Accordingly, the defect site was shown to be created by metal ions (Pb (II) or halide (I^−^) remaining in the transport layer and perovskite layer^17^.

Several methods have been initiated to improve the properties of the electron transfer layer, for example, element doping, surface modifications, and multilayer ETL structures^18^. Recent research has demonstrated that certain organic electron transfer layers can decrease the density of trap states at the grain boundaries, and on the surface of perovskite crystals, improving the efficiency of electron extraction and lowering optical photocurrent hysteresis^19–21^. Also, the polymer additives aid in raising the quality of the electron transfer materials, which reduces combination, and improves the photovoltaic parameters of PSCs. So, a simple and novel strategy to significantly increase the stability and PCE of PSCs is using polymer additives with the intention of fine tuning the overall morphology. The uniformity of the perovskite layer can be improved in the presence of the polymer additives by decelerating aggregation and the growth of the layer. Conductive polymers such as Phenyl-C61-butyric acid Methyl ester (PCBM) and polyaniline (PANI) can improve the charge transport properties^22^.

In this investigation, SnO_2_@ZnO nanocomposites were synthesized using *Calotropis* plant extract as a renewable reductant and mild stabilizer by a straightforward eco-friendly approach as electron transfer materials to create low-temperature-processed PSCs. For SnO_2_@ZnO nanocomposites, the PSCs had a comparatively high PCE of 11.18%. Notably, the SnO_2_@ZnO nanocomposite-based device demonstrated better stability in contrast to that based on TiO_2_. Polyaniline (PANI) is the most extensively used electrode material due to its simple synthesis, high electrical conductivity, environmental stability, and superior charge carrier mobility^23^. Therefore, SnO_2_@ZnO.PANI nanocomposites were synthesized for the first time in this study using a green, and simple route. Polyaniline polymer was successfully used as an addition to enhance perovskite quality. In other words, we introduce PANI as an effective polymeric additive in SnO_2_@ZnO ETM. Our research indicates that the addition of PANI helps to improve the perovskite film's quality, producing a device that kept 89% of its initial performance even after 480 h of operation in the air without any encapsulation.

## Experimental

### Materials and methods

The following is a list of the compounds utilized in this research, along with their manufacturers. Without additional purification, all of the components were used: Polyvinylpyrrolidone (PVP), zinc nitrate hexahydrate (Zn(NO_3_)_2_·6H_2_O), ammonium persulfate (APS), zinc powder (Zn), sodium perchlorate (NaClO_4_), with high purity were purchased from Sigma Aldrich. Aniline, chlorobenzene, hydrochloric acid (HCl, 37%), N,N-dimethylformamide (DMF, 99.9%), dimethyl sulfoxide (DMSO, 99.9%), acetonitrile, anhydrous ethanol (99.99%), and isopropanol (IPA, 99.7%) were purchased from Merck Chem. Co. (Germany). Lead (II) iodide (PbI_2_, 99.99%), Carbon paste, TiO_2_ paste (crystalline phase: anatase, particle size: 30 nm), methylammonium iodide (MAI, 99%), and fluorine tin oxide glass (FTO with 10–15 Ω/cm^2^) were obtained from Sunlab Co. (IRAN).

Wide-angle X-ray diffraction of the FTO/(SnO_2_@ZnO.PANI) nanocomposite/perovskite, and FTO/SnO_2_@ZnO nanocomposite/perovskite for structural analysis of these samples were recorded using XRD-Phillips X̕pert PRO with monochromatized Cu-Kα radiation (*λ* = 1*.*5178 Å). Fourier transform infrared (FT-IR) spectroscopy to determine the formation of ZnO@SnO_2_ nanocomposites, PANI nanoparticles (NPs), and SnO_2_@ZnO.PANI (2%, 4%, and 6%) nanocomposites were carried out using Tensor 27 spectrometer (Bruker, Saarbrucken, Germany). The morphological features of 4% SnO_2_@ZnO.PANI nanocomposites were investigated with a Zeiss (EM10C-Germany) transmission electron microscope (TEM) operating at 100 kV. The sample was ultrasonically dispersed in ethanol, and cast into carbon film on a copper grid 300 mesh (EMS-USA) for the measurement. The surface morphological features of perovskite films spin-coated on SnO_2_@ZnO nanocomposite, and 4% SnO_2_@ZnO.PANI nanocomposite were recorded using a field emission scanning electron microscopy (FE-SEM Sigma, Zeiss) equipped with energy dispersive X-ray (EDX) spectroscopy, and EDX-mapping analysis, the sample was prepared with gold coating for the measurement. UV–Vis spectra of SnO_2_@ZnO nanocomposites, PANI-NPs and SnO_2_@ZnO.PANI (2%, 4%, and 6%) nanocomposites dispersed in DMF under ultrasonic action were recorded on a SPECORD 210 (Analytic Jena, Germany). For determining the energy levels of SnO_2_@ZnO nanocomposites and SnO_2_@ZnO.PANI (2%, 4%, and 6%) nanocomposites, cyclic voltammetry (CV) measurements were carried out using an AutoLab device (302 N potentiostat, Netherlands). The electrochemical cell consisted of a conventional three-electrode configuration with two Pt electrodes as the counter, the working electrode, and an Ag/AgCl as the reference electrode in NaClO_4_ as the electrolyte has been used for this study. A 100 mV/s speed was used for the voltage sweep. A Solmetric I-V Curve Tracer (NanoSAT Co., Iran), and an Auto Adjustable Solar Simulator (Karmana Photonics, Iran, with an AM 1.5, 100 mW/cm^2^) were used to measure the photocurrent density–voltage (J–V) performance of the PSCs. A normative silicon cell was used to control the brightness of the light. An aperture was used to conceal the solar cells to create a 0.04 cm^2^ active area. Without any encapsulation, all measurements of the solar cells were made at room temperature, and in the natural atmosphere.

### Synthesis of nanomaterials

As previously described, a slightly modified technique was used to create SnO_2_@ZnO nanocomposites, PANI nanoparticles, and SnO_2_@ZnO.PANI nanocomposites^24,25^.

#### Green synthesis of SnO_2_@ZnO nanocomposites

Zn(NO_3_)_2_·6H_2_O (25 mL, 0.1 M) was added to SnCl_2_·2H_2_O (25 mL, 0.05 M), and the aqueous extract of the *Calotropis* (180 mL) for 30 min. The solution was centrifuged at 5000 rpm for 10 min after 8 h to remove sediments, and it was then rinsed three times with water before being dried at laboratory temperature. The SnO_2_@ZnO nanocomposites were produced after being heated to 550 °C for three hours in an electric oven.

#### Synthesis of PANI-NPs

The following was a typical PANI nanoparticle synthesis process: (1) To make solution A, 1.0 mL of concentrated HCl and 1.0 g of aniline monomers were dissolved in distilled water; (2) To make solution B, 0.8 mL of concentrated HCl, 2.85 g of ammonium persulfate (APS), and 2.0 g of PVP were dissolved in distilled water; (3) After precooling solution B for 30 min in an ice water bath, solution A was added within 2 h while being vigorously stirred; (4) The mixture was then stirred continuously for 24 h to complete the oxidative polymerization. Thereafter, the precipitated powder was filtered and washed with distilled water and ethanol until the filtrate became colorless. The precipitate was dried for 24 h in an oven at 70 °C. The dry powder could be re-dispersed in 0.1 M HCl in the ultrasonic bath for 30 min to ensure that PANI was adequately doped with HCl. It was then filtered, collected, and dried once again. PANI-NPs are the name given to the finished products.

#### Synthesis of SnO_2_@ZnO.PANI nanocomposite

**Figure 1:**
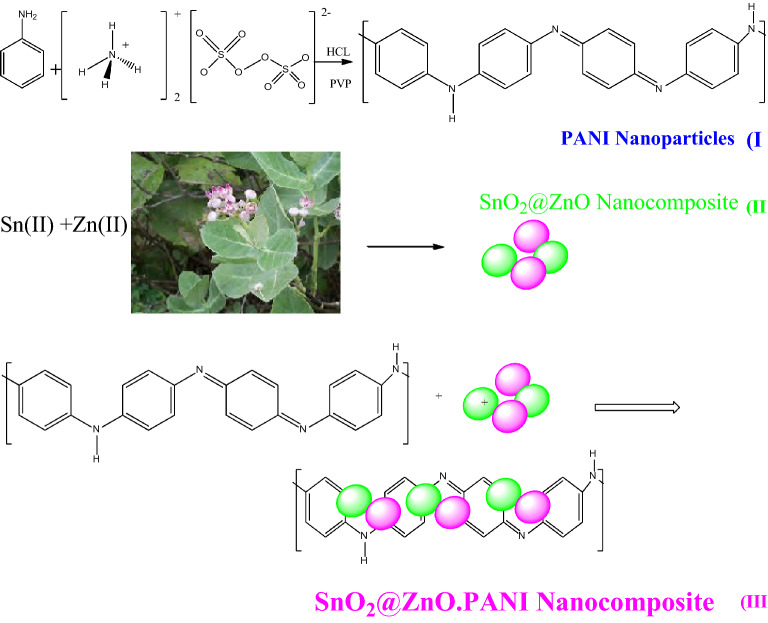
The green synthesis procedure of SnO_2_@ZnO.PANI nanocomposite.

The four different SnO_2_@ZnO.PANI nanocomposite samples were made using the following technique:

The generated PANI was diluted with DMF (0.013 g were dispersed into 30 mL DMF under stirring at 40 °C), and combined with SnO_2_@ZnO solution (0.09 g were dispersed into 30 mL DMF under stirring at 40 °C) in the ultrasonic bath at different volume ratios (0, 2, 4, and 6% v/v), which was followed by mixing at room temperature for 24 h and hand left for 48 h for polymerization. The sample was then dried for 24 h in an oven at 70 °C (Fig. 1).

### Device fabrication (assembly of solar cells)

For the comparison study, four different hole-transfer layer (HTL)-free-PSCs with an overall structure of FTO/ETL/CH_3_NH_3_PbI_3_/C-paste were designed. In all four-model construction, the processes and the depositing of all layers are the same except for the ETL. The steps for making these cells are bellowed:

Before assembling the solar cells, to get the required pattern, the FTO glass was etched using zinc powder, and 2.0 M HCl aqueous solution, then ultrasonically cleaned with deionized water, isopropanol, acetone, and anhydrous ethanol. For the preparation of the FTO/c-TiO_2_, titanium diisopropoxide bis (acetylacetonate) in anhydrous ethanol (1:10 (v/v)) was formed by spin-coating on the FTO glass at 4500 rpm for 45 s and then heated at 500 °C for 30 min. Then, FTO/c-TiO_2_/m-TiO_2_ ETL was made by spin-coating TiO_2_ paste diluted in anhydrous ethanol at a ratio of 1:5 spin-coated at 4500 rpm for 30 s and then sintered in air at 500 °C for 30 min. For FTO/SnO_2_@ZnO.PANI (0%, 2%, 4%, and 6%), ETL of SnO_2_@ZnO.PANI (0%, 2%, 4%, and 6%) nanocomposites solution was prepared in anhydrous ethanol and spin-coated at 4500 rpm for 30 s followed by annealing at 110 °C in the air for 30 min to remove the H_2_O between the molecules in the film. For FTO/TiO_2_/SnO_2_@ZnO and FTO/TiO_2_/SnO_2_@ZnO.PANI ETL, the SnO_2_@ZnO and the best volume ratios of SnO_2_@@ZnO.PANI 4% nanocomposites employed on top of the c-TiO_2_ ETL, respectively. To prepare the active layer, the deposition occurs in two steps. In the first step, 461 mg of PbI_2_ dissolved in 1.0 mL DMF and DMSO as a mixed solvent 9:1 (v/v) and was spin-coated on ETL substrate at 4000 rpm for 20 s, and then the PbI_2_ film was annealed at 70 °C for 2 min. During the deposition, 200 μL of chlorobenzene was dripped onto the substrates 10 s before to the end of the program act as an anti-solvent. In the second step, 30 mg/mL of MAI-IPA solution was prepared, and the cells were immersed in it for 5 min. Subsequently, the substrate containing the perovskite layer was heated at 80 °C for 15 min after deposition. Finally, using a Doctor-Blade approach, the carbon back-electrode was coated onto the perovskite layer with commercial carbon paste, and the complete device was cured at 100 °C for 30 min.

## Results and discussion

### XRD analysis

**Figure 2:**
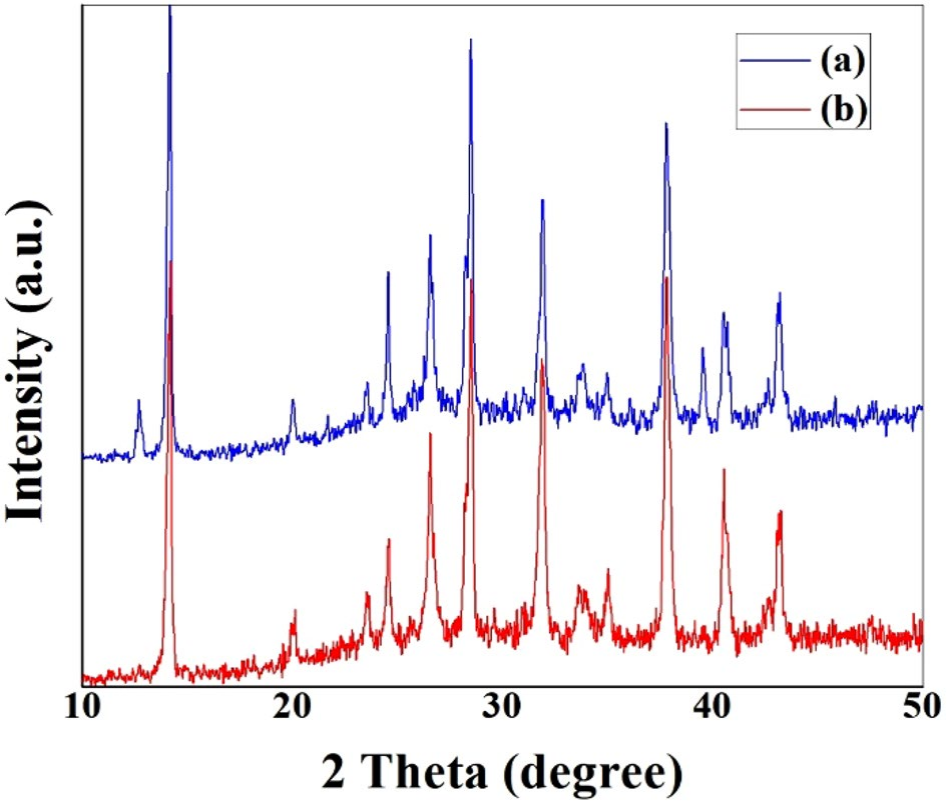
XRD patterns of (**a**) FTO/SnO_2_@ZnO nanocomposite/perovskite, and (**b**) FTO/SnO_2_@ZnO.PANI nanocomposite/perovskite.

The generated perovskites' XRD patterns, as shown in Fig. 2, indicate identical crystal structures with distinctive peaks at 2θ = 14.19°, 28.51°, and 31.93°, which correspond to the (101), (202), and (211) planes, respectively^26,27^. Additionally, the fact that the perovskite spin-coated on SnO_2_@ZnO.PANI nanocomposite layer considerably increases the intensity of the (101) peak, suggesting that crystals develop more effectively. The 4% PANI additive reduced the full width at half maximum (FWHM) of the main peak at 14.19° from 0.118 to 0.098, while the peak's position remained unchanged. This means that the perovskite structure has a better molecular ordering. Therefore, a better-ordered grain orientation for perovskite film (CH_3_NH_3_PbI_3_) was created by SnO_2_@ZnO.PANI nanocomposite, and a preferred growth along (101) diffraction facet was assessed. The PbI_2_ cubic structure, created during the annealing of perovskites, is responsible for the detectable signal's presence at 12.7°^8^. The appearance of a PbI_2_ peak in a SnO_2_@ZnO nanocomposite suggests that there is still PbI_2_ present, whereas the PbI_2_ peak is removed with PANI modification.

### FT-IR analysis

**Figure 3:**
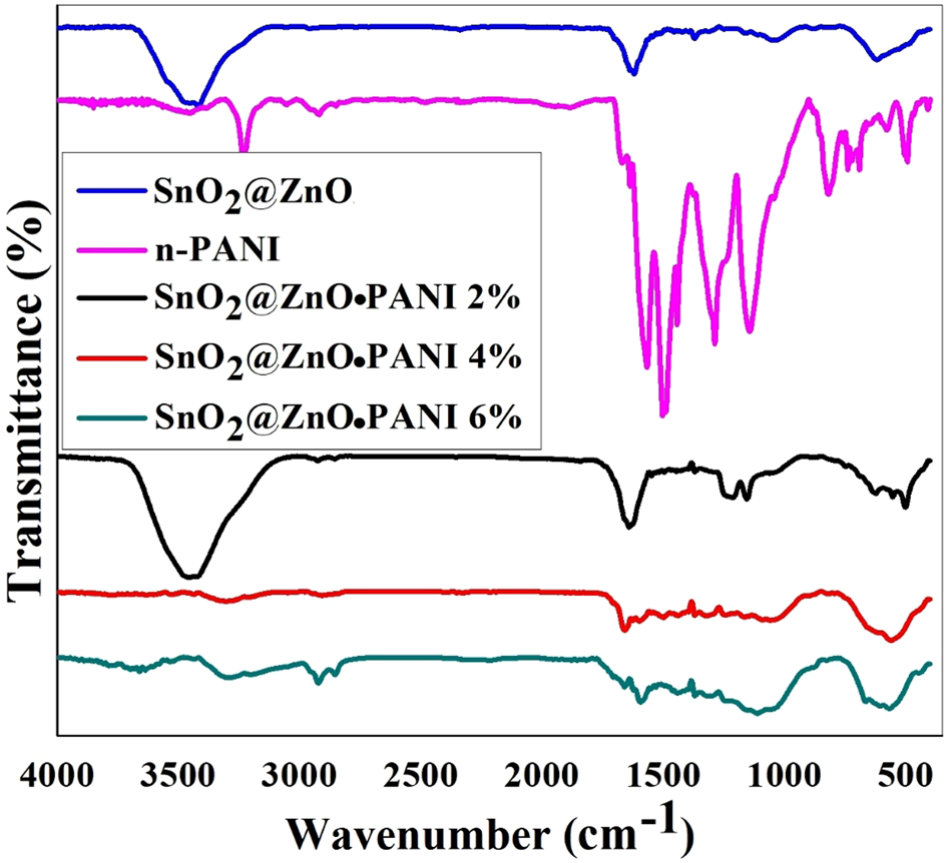
FT-IR spectra for SnO_2_@ZnO nanocomposite, PANI-NPs and different volume ratios of SnO_2_@ZnO.PANI (2%, 4%, and 6%) nanocomposites.

The chemical structure of the products was characterized by FT-IR spectroscopies and shown in Fig. 3. According to the results, the FT-IR spectrum for the SnO_2_@ZnO nanocomposite displayed in Fig. 3, two new bands around 620 and 500 cm^−1^ are attributed to SnO_2_ and ZnO, respectively. The bands at 3417 cm^−1^ and 1621 cm^−1^ are due to humidity adsorption by the nanocomposite^28^.

On the other hand, for the PANI-NPs in Fig. 3, the strong peaks in wave number range of 1000–1600 cm^−1^, attributed to the vibrational bands. The main bands at 1567 cm^−1^ and 1503 cm^−1^ were attributed to C=N and C=C stretching modes of vibration for the quinonoid and benzenoid units of PANI. The peaks around 1288 cm^−1^ are assigned to C–N stretching mode of the benzenoid ring^29–31^. The band at 692 cm^−1^ can be assigned to aromatic C–H out-of-plane bending vibrations. The band at 1144 and 819 cm^−1^ is due to the aromatic C–H in-plane bending and the out-of-plane deformation of C–H in the 1,4-disubstituted benzene ring. The small peak at 3235 cm^−1^ corresponds to the presence of secondary amine stretching (N–H) vibrations^30^. The band at 494 cm^−1^ is due to C–H out-of-plane bending vibration^31^.

Also, the FT-IR spectroscopy of SnO_2_@ZnO.PANI nanocomposites is used to study the chemical interaction. Figure 3 shows FT-IR spectra of all the samples SnO_2_@ZnO.PANI nanocomposites at different volume ratios, which shows some slight shift in the wave number as well as change in the intensity of peaks as compared to PANI and SnO_2_@ZnO nanocomposite. For all different volume ratios of SnO_2_@ZnO.PANI nanocomposite, peaks observed around 1595 cm^−1^ and 1444 cm^−1^ are attributed to (C=N) and (C=C) stretching mode of vibration for the quinonoid and the benzenoid units of SnO_2_@ZnO.PANI nanocomposites. The peak around 1113 cm^−1^ is assigned to C–N stretching mode of benzenoid ring^31–33^. The bands around 620 and 567 cm^−1^ are due to the presence of SnO_2_ and, ZnO, respectively in the nanocomposite^34,35^. Thus FT-IR peak results indicate that some interactions exist between SnO_2_@ZnO nanocomposite, and PANI nanoparticles, and the formation of SnO_2_@ZnO.PANI nanocomposites occurred in the PANI matrix.

### TEM analysis

**Figure 4:**
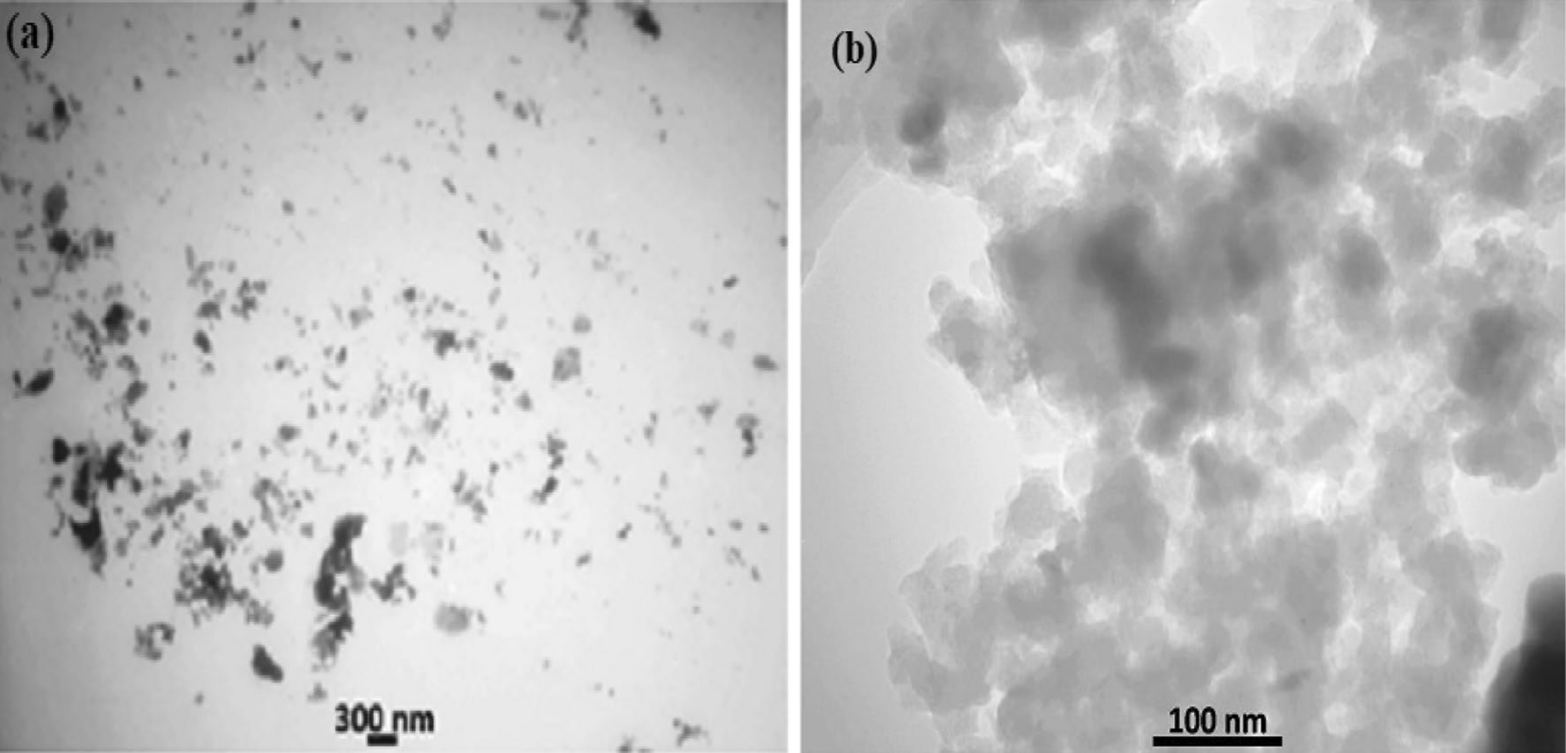
TEM images of (**a**) SnO_2_@ZnO nanocomposites and (**b**) SnO_2_@ZnO.PANI 4% nanocomposite.

The structures of SnO_2_@ZnO and SnO_2_@ZnO.PANI 4% nanocomposite were further investigated by TEM analysis. According to Fig. 4, the morphology of the SnO_2_@ZnO nanocomposites is spherical.

It can be confirmed that SnO_2_@ZnO nanocomposites were embedded in PANI-NPs (Fig. 4b). It can be seen the ultra-fine particles of the transition metal oxides are in the nanometer range.

### FE-SEM, EDX, and EDX mapping analysis

**Figure 5:**
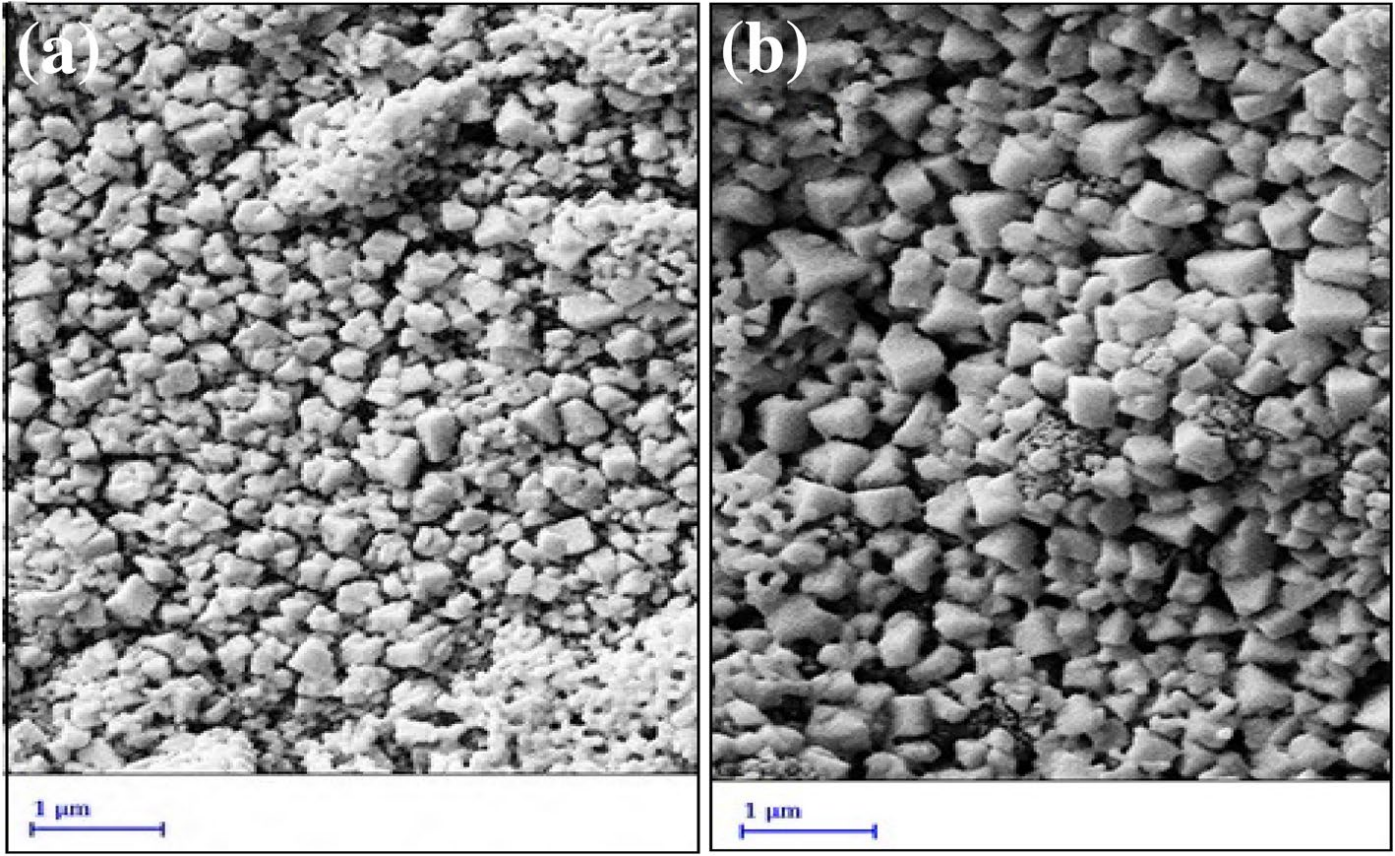
Top-view SEM images of the MAPbI_3_ perovskite films deposited on (**a**) SnO_2_@ZnOnanocomposite and (**b**) SnO_2_@ZnO.PANI 4% nanocomposite.

It's crucial to provide a perovskite film with big, uniform grains. Additionally, it is well known that CH_3_NH_3_PbI_3_ film readily deteriorates in an air-conditioned environment due to grain boundaries moisture penetration. Therefore, improving perovskite morphology is another effective way to lessen CH_3_NH_3_PbI_3_ film instability^8^. According to Fig. 5a,b, FE-SEM is used to analyze the morphology of the perovskites film that deposited on SnO_2_@ZnO nanocomposite, and SnO_2_@ZnO.PANI nanocomposite as ETM. It is clear that the perovskite grain size grows when the PANI add to SnO_2_@ZnO, and the perovskite surface becomes more homogenous. This suggests that a higher-quality perovskite layer is produced when a proper amount of PANI is included in the SnO_2_@ZnO as ETM. The PANI acts as a scaffold for crystallization of the perovskite layer, and improves the perovskite photovoltaic performance and stability, attributed to the favorable interfacial interaction between –NH_2_^+^ groups of PANI and I of MAPbI_3_ perovskite^36^. They can passivate charge traps on the grain boundaries and act as an electron transfer channel to ETL^22^.

**Figure 6:**
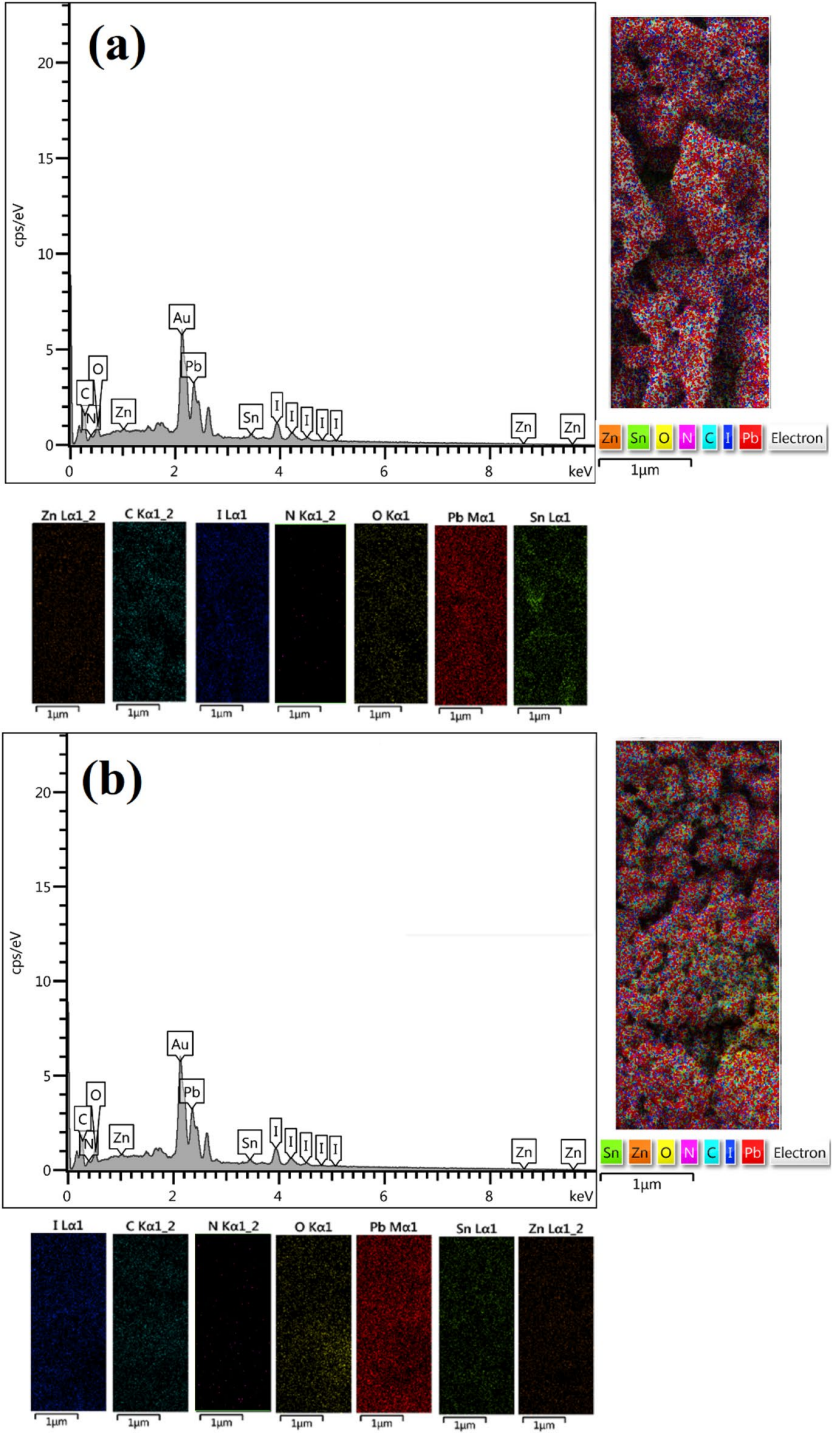
The EDX spectra and the mapping analysis for perovskite films spin-coated on (**a**) SnO_2_@ZnO nanocomposite, and (**b**) SnO_2_@ZnO.PANI 4% nanocomposite.

EDX spectroscopy has been performed for the elemental study of the perovskite film deposited on SnO_2_@ZnO, and the SnO_2_@ZnO.PANI nanocomposite layer. All the inorganic elements of CH_3_NH_3_PbI_3_ have been found with projected composition shown by Fig. 6a,b.

EDX-mapping investigation was also used, and the results are exhibited in Fig. 6a,b. The mapping analysis confirmed the formation of perovskite film on nanocomposites, as well. It also showed that the two main elements of Pb and I are well distributed on a large scale, confirming the uniformity of the MAPbI_3_ film grown on the nanocomposites structure^37^.

### UV–visible analysis

**Figure 7:**
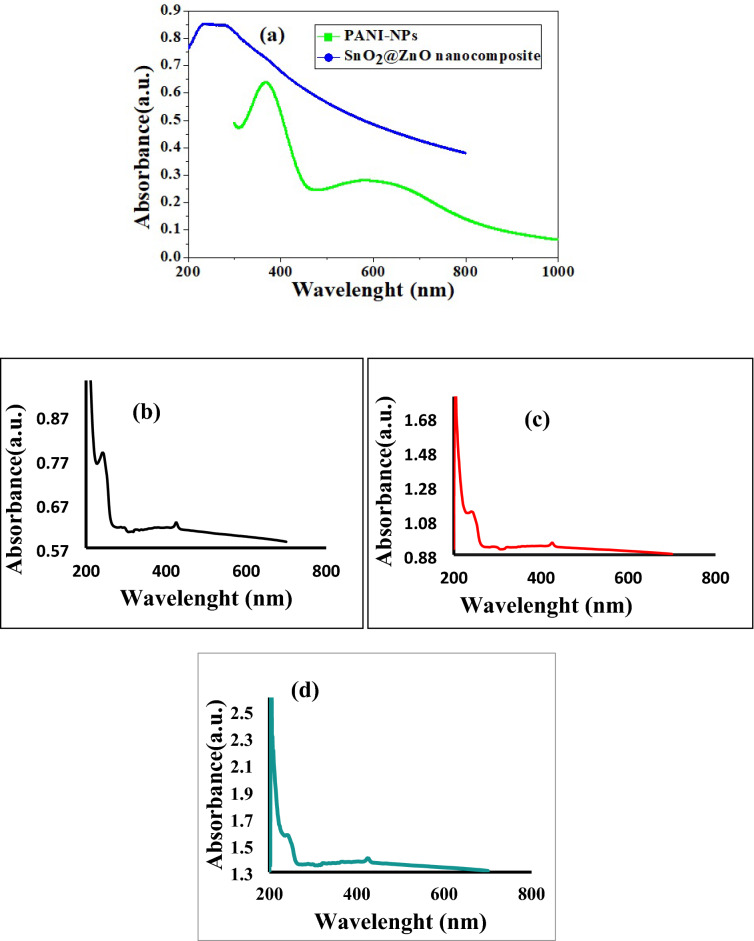
UV–Vis absorption spectra of (**a**) SnO_2_@ZnO nanocomposite (blue) and PANI-NPs (green) and (**b**–**d**) different volume ratios of SnO_2_@ZnO.PANI (2%, 4%, and 6%) nanocomposite.

**Figure 8:**
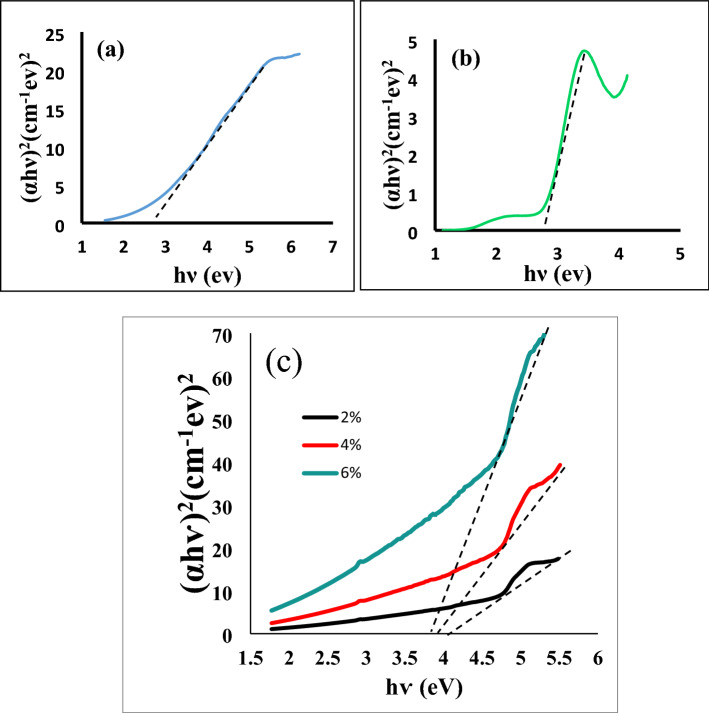
Tauc plots (**a**–**c**) showing indirect band gap values of (**a**) SnO_2_@ZnO nanocomposite, (**b**) PANI-NPs (**c**) different volume ratios of SnO_2_@ZnO.PANI (2%, 4%, and 6%) nanocomposite.

Optical absorption spectrum for SnO_2_@ZnO nanocomposite, PANI-NPs, and SnO_2_@ZnO.PANI (2%, 4%, and 6%) nanocomposites in spectral series of 200–800 nm was recorded by using ultraviolet–visible spectroscopy and shown in Fig. 7a–d. To obtain complete information about the energy gap for the samples, an examination of the dependency of the absorption coefficient on photon energy in the high absorption area was done. The optical band gaps (Eg) are evaluated based on Tauc's relation ${(\alpha \mathrm{h}\nu )}^{\frac{1}{\mathrm{r}}}=\mathrm{A}\left[\mathrm{h}\nu -{E}_{g}\right],$ where A is a constant, $\nu$ is the frequency, h is the Planck constant, *α* is the light absorption index, and *E*_*g*_ is the band gap of the semiconductor, respectively. The value of r is dependent on optical absorption; for example, 1/2 and 2 are presented as the direct allowed and indirectly allowed transitions, respectively. Figure 8 illustrates the graph between (αhv)^2^ and (hv) in eV, to determine the energy bandgap. The band gaps of SnO_2_@ZnO nanocomposite (a), PANI-NPs (b) and different volume ratios of SnO_2_@ZnO.PANI (2%, 4%, and 6%) nanocomposite (c) were determined to be 2.82, 2.76, 4.05, 3.97 and 3.88 eV respectively^38^.

### Electrochemical measurements

**Figure 9:**
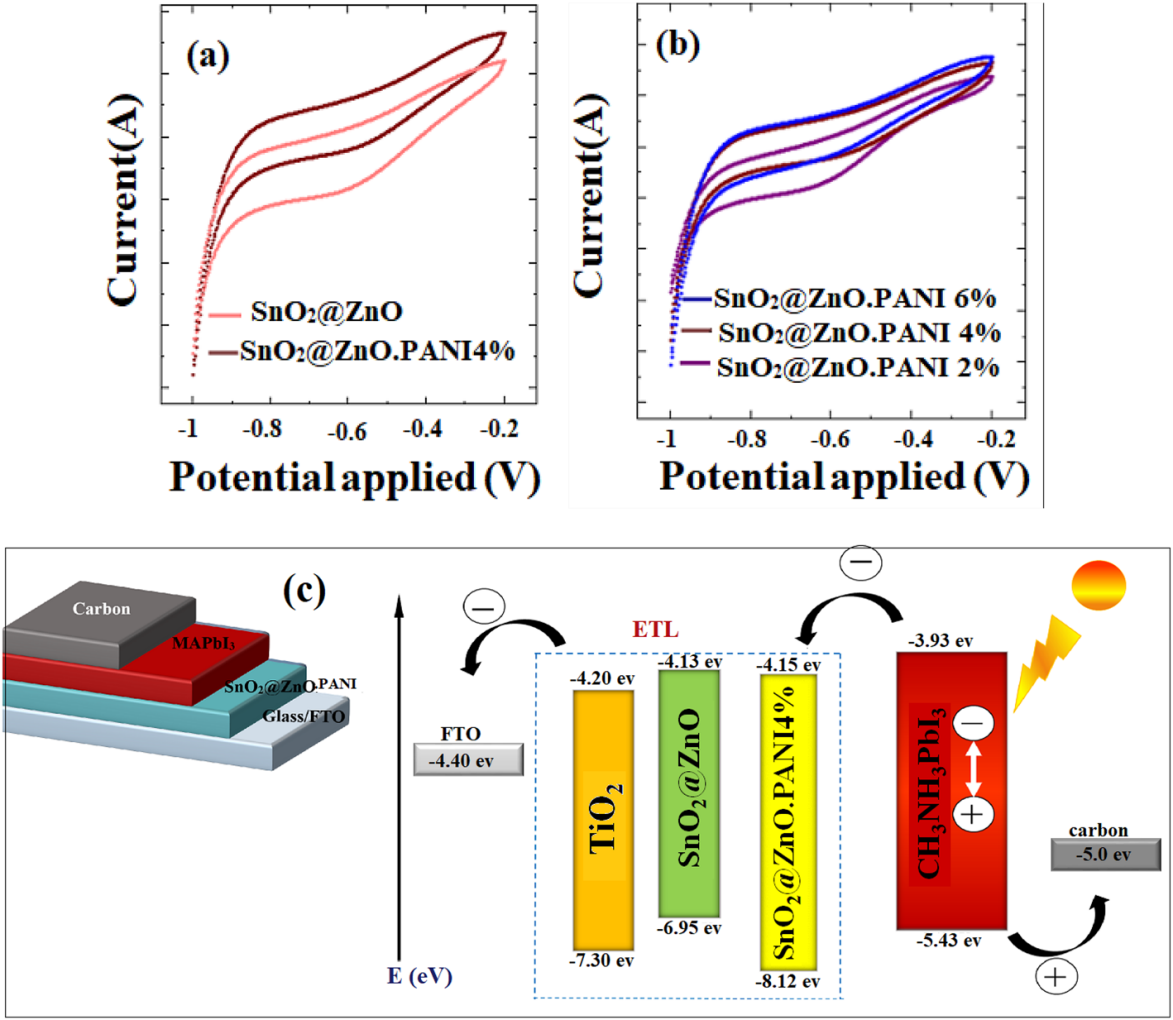
Cyclic voltammetry graphs of (**a**) SnO_2_@ZnO nanocomposite and SnO_2_@ZnO.PANI 4% nanocomposite, (**b**) different volume ratios of SnO_2_@ZnO.PANI (2%, 4%, and 6%) nanocomposite, and (**c**) Energy level diagram for all materials used in PSCs fabrication.

Cyclic voltammetry was carried out to determine the electrochemical properties, such as energy levels of synthesis materials. Figure 9a,b presents the CV graphs of synthesized nanocomposites. The following equation can be used to calculate the lowest unoccupied molecular orbital (LUMO) level of the materials^39^:

$$ {\text{E}}_{{{\text{LUMO}}}} \, = \, - \left( {{\text{E}}_{{{\text{red}}}} \, + \,{4}.{75}} \right){\text{ eV}}. $$

The highest occupied molecular orbital (HOMO) energy can be obtained using E_HOMO_ = E_LUMO_ − Eg. Using the values obtained by the Tauc-plot and cyclic voltammetry, it was possible to obtain the energy levels for SnO_2_@ZnO nanocomposite and different volume ratios of SnO_2_@ZnO.PANI nanocomposite and correlate them with energy levels of the CH_3_NH_3_PbI_3_ and TiO_2_ mesoporous layers. LUMO and HOMO energy levels of these materials calculated from cyclic voltammograms are listed in Table 1.

**Table 1:** Optical and electrochemical properties of SnO_2_@ZnO nanocomposite, different volume ratios of SnO_2_@ZnO.PANI (2%, 4%, and 6%) nanocomposite and TiO_2_.

	0%	2%	4%	6%	TiO_2_
Eg (eV)	2.82	4.05	3.97	3.88	3.10
LUMO (eV)	− 4.13	− 4.115	− 4.15	− 4.125	− 4.20
HOMO (eV)	− 6.95	− 8.165	− 8.12	− 8.005	− 7.30

Electrons transferred from the perovskite are achieved using LUMO levels of the ETMs, estimating values between − 4.15 to − 4.115 eV from the first reduction peak in the CV curves of nanocomposites (Fig. 9). As the conduction band of perovskite CH_3_NH_3_PbI_3_ used in this work is − 3.93 eV, illustrating that the investigated nanocomposite compounds own favorable energetics for electron extraction.

Based on these results, Fig. 9c shows the energy level diagram for the materials used in PSCs fabrication, where all the PSCs were fabricated with a device configuration of FTO/ETL/perovskite layer/C.

**Figure 10:**
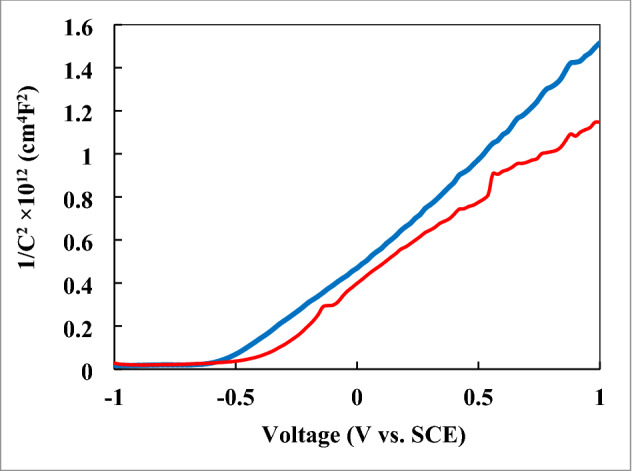
The MS plot for n-type SnO_2_@ZnO nanocomposite (blue line) and SnO_2_@ZnO.PANI 4% nanocomposite (red line) (electrolyte: Na_2_SO_4_ 0.5 M, Frequency: 1 kHz).

The type of semiconductors made was obtained by Mott–Schottky (M–S) method. First, the Mott–Schottky analysis (1/C^2^ versus voltage) was considered to calculate the density of charge carriers (concentration of charge carriers) and the flat band potential (VFB) according to M–S Eq. (1)^40,41^:

1 $$ \frac{1}{{{\text{C}}^{2} }} = \frac{2}{{{{\text{q}}\upvarepsilon_{{0}}} {\upvarepsilon }A^{2} {\text{N}}_{{\text{D}}} }}\left( {{\text{V}}_{{\text{FB }}} - {\text{V}} - { }\frac{{{\text{K}}_{{\text{B }}} {\text{T}}}}{{\text{q}}}} \right), $$

where q, ε_0_, ε, A, N_D_, V, V_FB_, K_B_, and T are the elementary charge, the vacuum permittivity, the dielectric constant of the compound, the surface area, the acceptor density, the applied potential, the flat band potential, the Boltzmann constant, and the absolute temperature. Figure 10 shows the Mott Schottky plot for the SnO_2_@ZnO nanocomposite and SnO_2_@ZnO.PANI (4%) nanocomposite. The positive slope in the linear region indicated that the SnO_2_@ZnO nanocomposite and SnO_2_@ZnO.PANI (4%) nanocomposite are N-type semiconductors.

**Figure 11:**
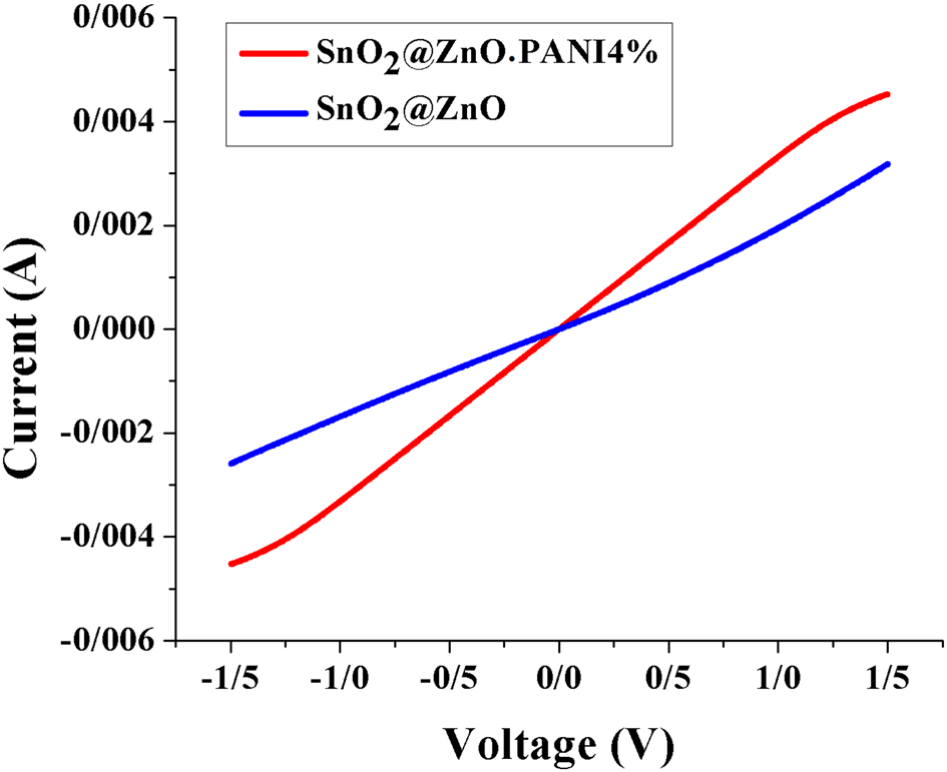
I–V curves of the device with glass/FTO/ETLs/carbon structure.

Conductivity is a critical parameter for the ETLs, so we have fabricated FTO/ETLs/carbon, and current density–voltage curves measured in the dark (Fig. 11). The I–V plot becomes steeper after loading the PANI additive. The SnO_2_@ZnO.PANI (4%) nanocomposite revealed a higher I–V plot than pure SnO_2_@ZnO nanocomposite film. The conductivity (σ) of the ETM improved from 8.5 × 10^–7^ S/cm to 3.8 × 10^–6^ S/cm by adding PANI additive. The finding of increased σ is in accordance with the photovoltaic parameters of PSCs, that the high conductivity of the ETL is an essential factor in improving FF and Jsc due to can lower the contact resistance and facilitate the carrier transfer.

### Photovoltaic properties of PSCs achieved via ETL modification

**Figure 12:**
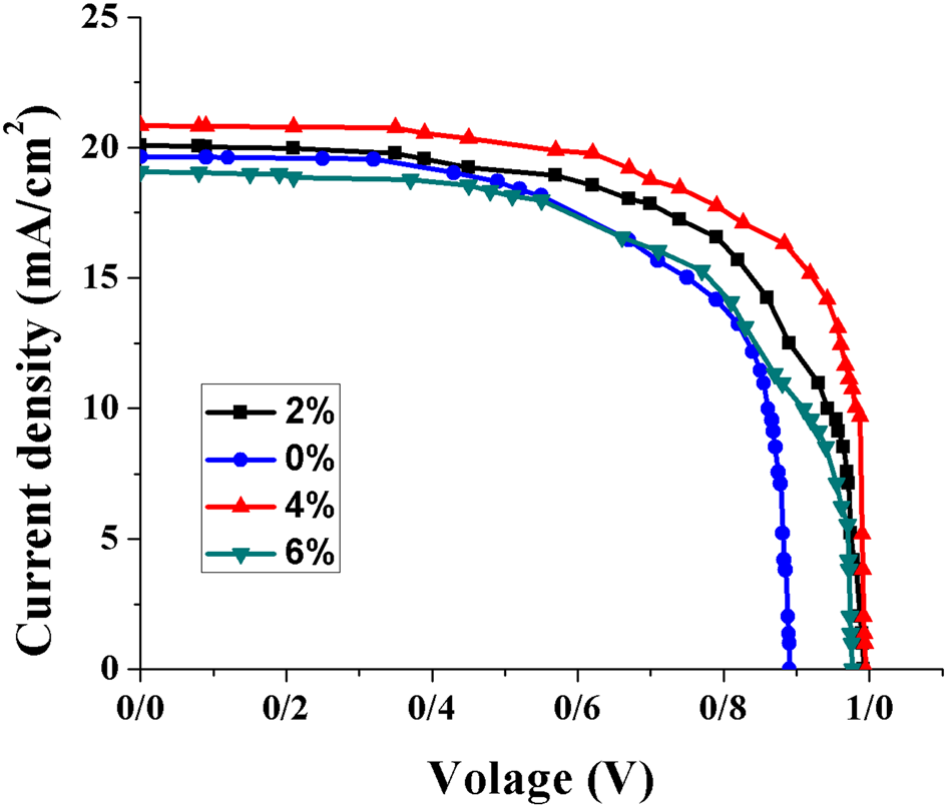
The J–V tests of the PSCs from different SnO_2_@ZnO.PANI (0%, 2%, 4%, and 6%) nanocomposite concentration as ETL.

First, to clarify the benefits of PANI additives, we constructed devices of FTO/ SnO_2_@ZnO.PANI/perovskite/carbon with the SnO_2_@ZnO.PANI nanocomposite with different volume ratios. The photovoltaic parameters of the perovskite solar cells were measured through the J-V curves under AM 1.5G illumination, as shown in Fig. 12 and Table 2.

**Table 2:** Photovoltaic performance of PSCs containing SnO_2_@ZnO.PANI (0%, 2%, 4%, and 6%) nanocomposite as ETL.

Device	Cell configuration	J_SC_ (mA/cm^2^)	V_OC_ (V)	FF	ƞ (%)
0%	FTO/SnO_2_@ZO nanocomposite/Perovskite/C	19.64	0.89	0.64	11.18
2%	FTO/SnO_2_@ZnO.PANI 2% nanocomposite/Perovskite/C	20.07	0.992	0.65	12.94
4%	FTO/SnO_2_@ZnO.PANI 4% nanocomposite/Perovskite/C	20.84	0.995	0.69	14.3
6%	FTO/SnO_2_@ZnO.PANI 6% nanocomposite/Perovskite/C	19.08	0.976	0.63	11.73

The efficiency of solar cells, η, is calculated by Eq. (2)^42^:

2 $$ \eta = {\text{P}}_{{{\text{max}}}} /{\text{P}}_{{{\text{in}}}} = {\text{J}}_{{{\text{SC}}}} \times {\text{V}}_{{{\text{OC}}}} \times {\text{FF}}/{\text{P}}_{{{\text{in}}}} , $$

where V_OC_, J_SC_, P_in_, and FF are the open circuit photovoltage, short circuit photocurrent density, power of white light, and fill factor of the cell, respectively. According to Eq. (3), the fill factor is defined by the ratio of the full power (P_max_ = J_opt_ × V_opt_) of the solar cell per unit area to V_OC_ and J_SC_:

3 $$ {\text{FF}} = {\text{J}}_{{{\text{opt}}}} \times {\text{V}}_{{{\text{opt}}}} /{\text{J}}_{{{\text{SC}}}} \times {\text{V}}_{{{\text{OC}}}} , $$

where J_opt_ and V_opt_ are the current density and voltage for determination of the power output, respectively, when PANI polymer was used, PSCs showed improved PCE, which is directly affected by the percentage of PANI additive. According to the obtained result, when the device was fabricated with 4% PANI the highest performance of PCE 14.3 was achieved. Based on this observation, 4% PANI is suggested as the optimum amount of SnO_2_@ZnO.PANI nanocomposite as ETL. These improvements in the photovoltaic parameters resulted from suppression in non-radiative recombination, the larger grain size of the MAPbI_3_, and grain boundaries’ inactivation.

**Figure 13:**
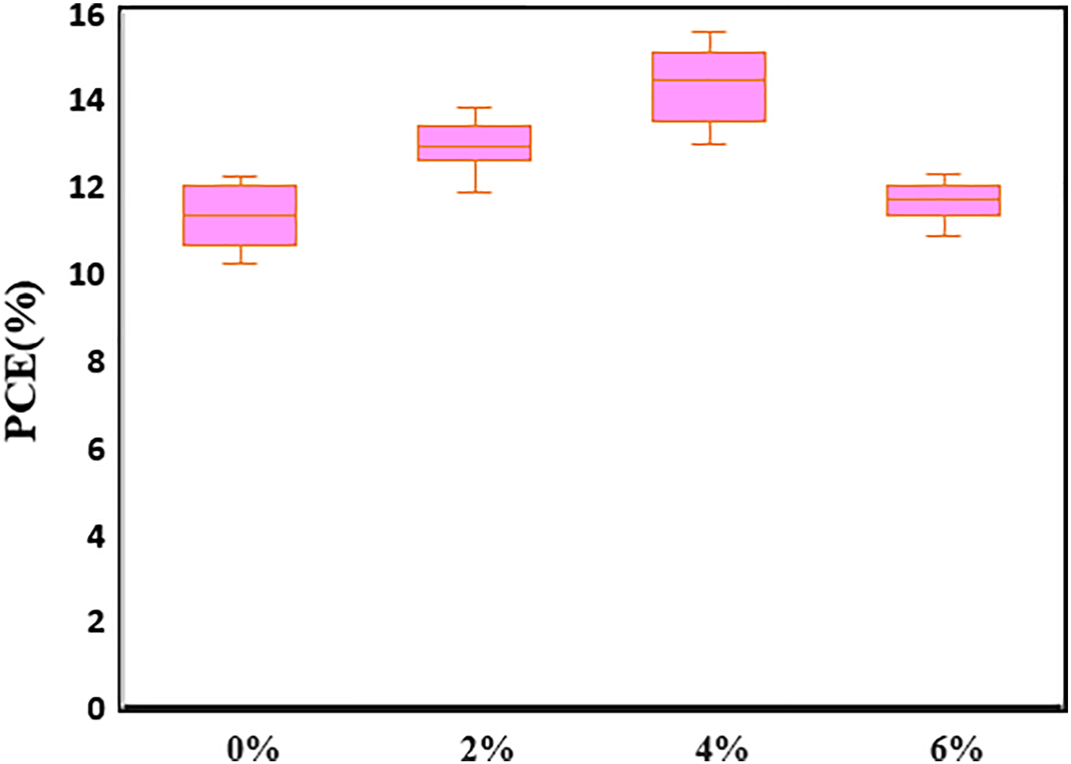
PCE histogram of 32 independent PSCs based on SnO_2_@ZnO.PANI (0%, 2%, 4%, and 6%) nanocomposite as ETL.

Furthermore, the reproducibility of the PCE data was studied, including 32 devices. Figure 13 displays the histograms of PCE values.

**Figure 14:**
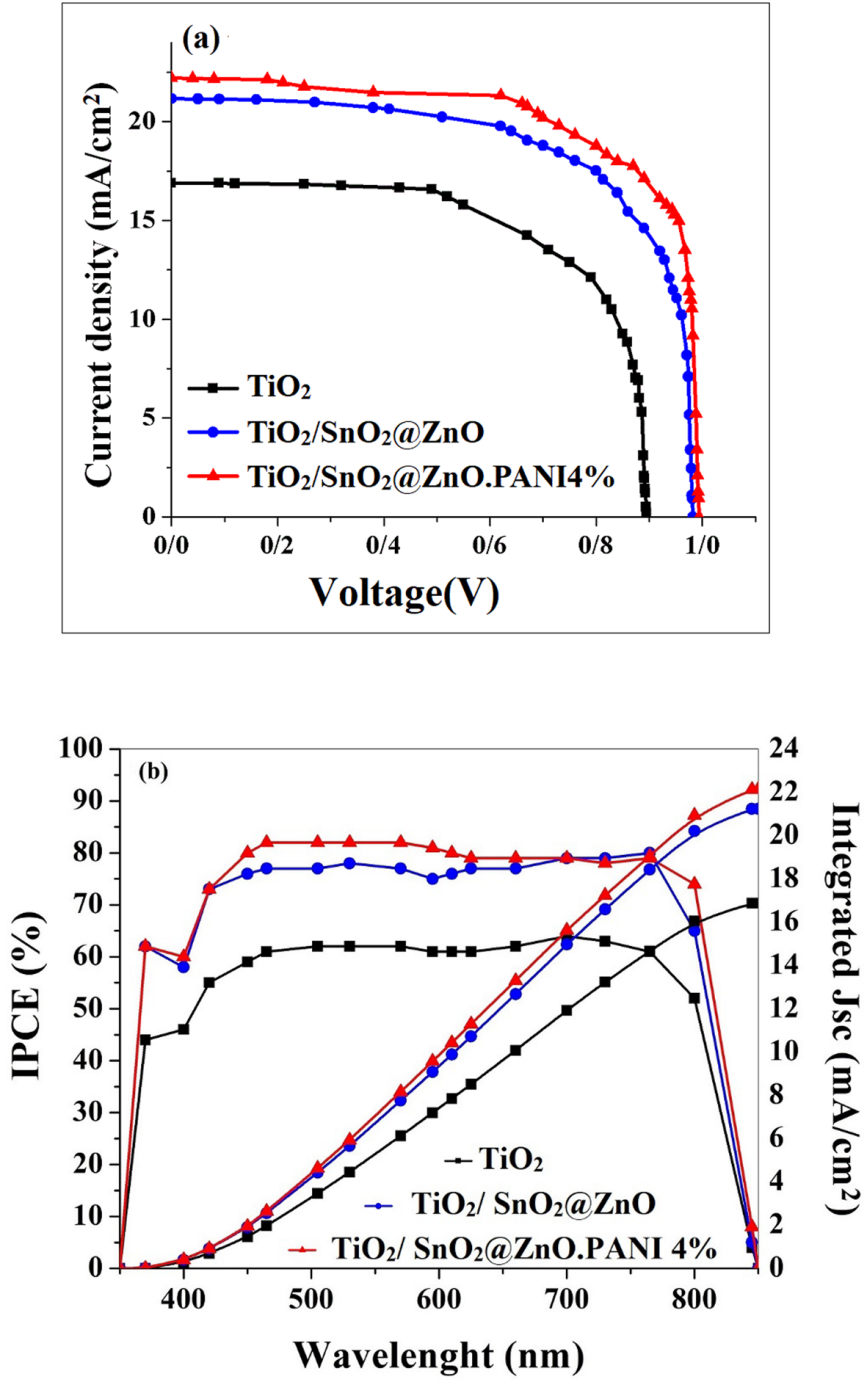
(**a**) The J–V tests of the PSCs from TiO_2_/SnO_2_@ZnO.PANI 4% nanocomposite, TiO_2_/SnO_2_@ZnO/nanocomposite, and TiO_2_ as ETL, and (**b**) The IPCE spectra of different PSCs based on TiO_2_/m-TiO_2_, TiO_2_/SnO_2_@ZnO nanocomposite and TiO_2_/SnO_2_@ZnO.PANI 4% nanocomposite as ETL, and the integrated current densities from the IPCE.

**Figure 15:**
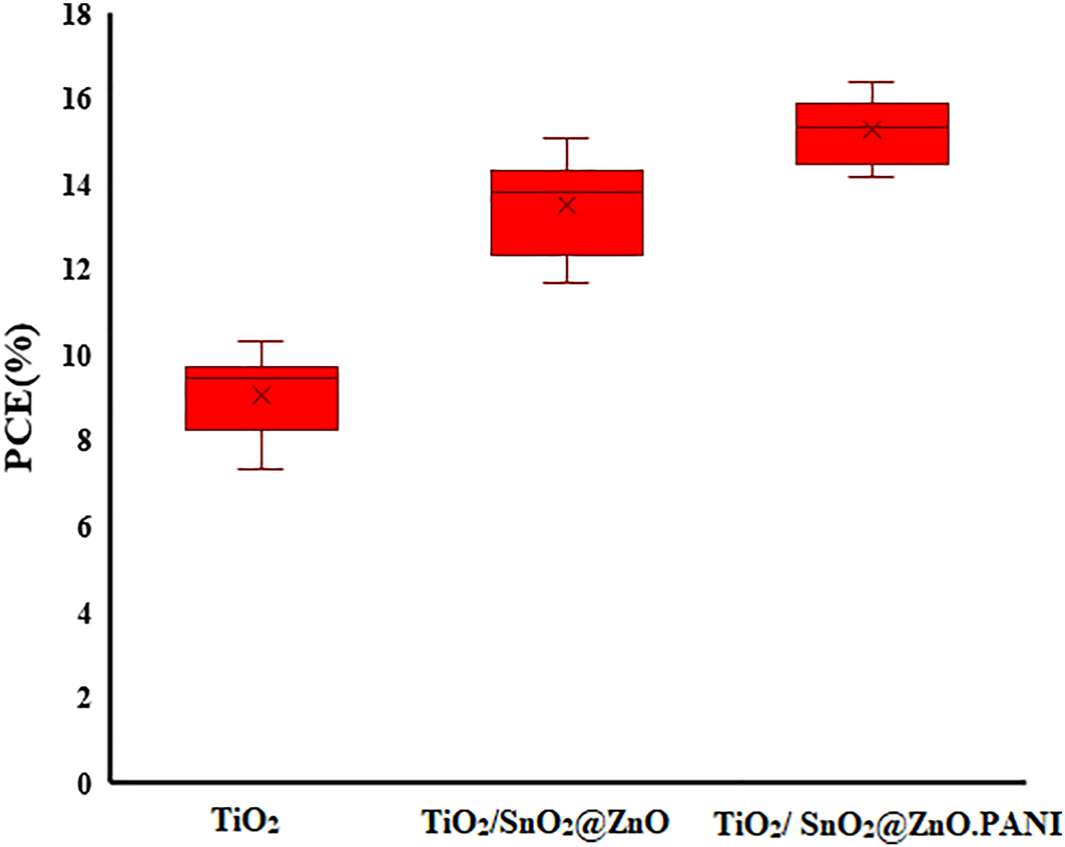
PCE histogram of 30 independent PSCs based on TiO_2_, TiO_2_/SnO_2_@ZnO nanocomposite, and TiO_2_/ SnO_2_@ZnO.PANI 4% nanocomposite as ETL.

After the optimum volume ratio of the SnO_2_@ZnO.PANI nanocomposite as ETM layer was found, PSCs based on the optimum volume ratio of the nanocomposite, devices of FTO/TiO_2_/SnO_2_@ZnO.PANI 4%/perovskite/carbon, FTO/TiO_2_/SnO_2_@ZnO/perovskite/carbon, and FTO/TiO_2_/perovskite/carbon were fabricated. Figure 14 depicts the J–V curves of various devices, and Table 3 also summarizes the PV characteristics of these PSCs. These experiments also suggest that the TiO_2_/SnO_2_@ZnO.PANI nanocomposite bilayer ETL is better than the TiO_2_ and SnO_2_@ZnO.PANI nanocomposite monolayer ETLs. To discuss the differences in photovoltaic performance for different ETLs, 30 pieces of PSCs were prepared to analyze the results under an AM 1.5 solar simulator Fig. 15. It is expected that improving the electron extraction and revising the band alignment of the TiO_2_/SnO_2_@ZnO.PANI nanocomposite bilayer ETL could increase the PEC in the PSCs. As expected, the TiO_2_/SnO_2_@ZnO.PANI 4% nanocomposite devices show a champion PCE of 15.45%; in this regard, the TiO_2_/SnO_2_@ZnO and TiO_2_ devices exhibit a PCE of 13.92% and 9.52%, respectively. The electrical conductivity of SnO_2_@ZnO.PANI 4% is better than SnO_2_@ZnO nanocomposite. Thus, the Jsc is improved significantly for SnO_2_@ZnO.PANI 4% PSCs, compared with SnO_2_ZnO PSC.

**Table 3:** Photovoltaic parameters of perovskite solar cell.

Cell configuration	J_SC_ (mA/cm^2^)	V_OC_ (V)	FF	ƞ (%)
FTO/c-TiO_2_/m-TiO_2_/Perovskite/C	16.9	0.895	0.63	9.52
FTO/ c-TiO_2_/SnO_2_@ZnO nanocomposite/Perovskite/C	21.16	0.982	0.67	13.92
FTO/c-TiO_2_/ SnO_2_@ZnO.PANI 4% nanocomposite/Perovskite/C	22.21	0.994	0.7	15.45

Besides, the spectra of the monochromatic incident photon to current efficiencies (IPCEs) of PSCs based on pure FTO/c-TiO_2_/m-TiO_2_, FTO/c-TiO_2_/SnO_2_@ZnO nanocomposite, and FTO/c-TiO_2_/ SnO_2_@ZnO.PANI 4% nanocomposite as ETL were measured to elucidate the J_SC_ improvement, and shown in Fig. 14b. The value of the integrated J_SC_ obtained from IPCE was 16.88 mA/cm^2^, 21.24 mA/cm^2^, and 22.14 mA/cm^2^ for control, and c-TiO_2_/SnO_2_@ZnO nanocomposite and c-TiO_2_/SnO_2_@ZnO.PANI 4% nanocomposite devices, respectively. These values agreed with the J_SC_ calculated from J–V measurements (Table 3).

**Figure 16:**
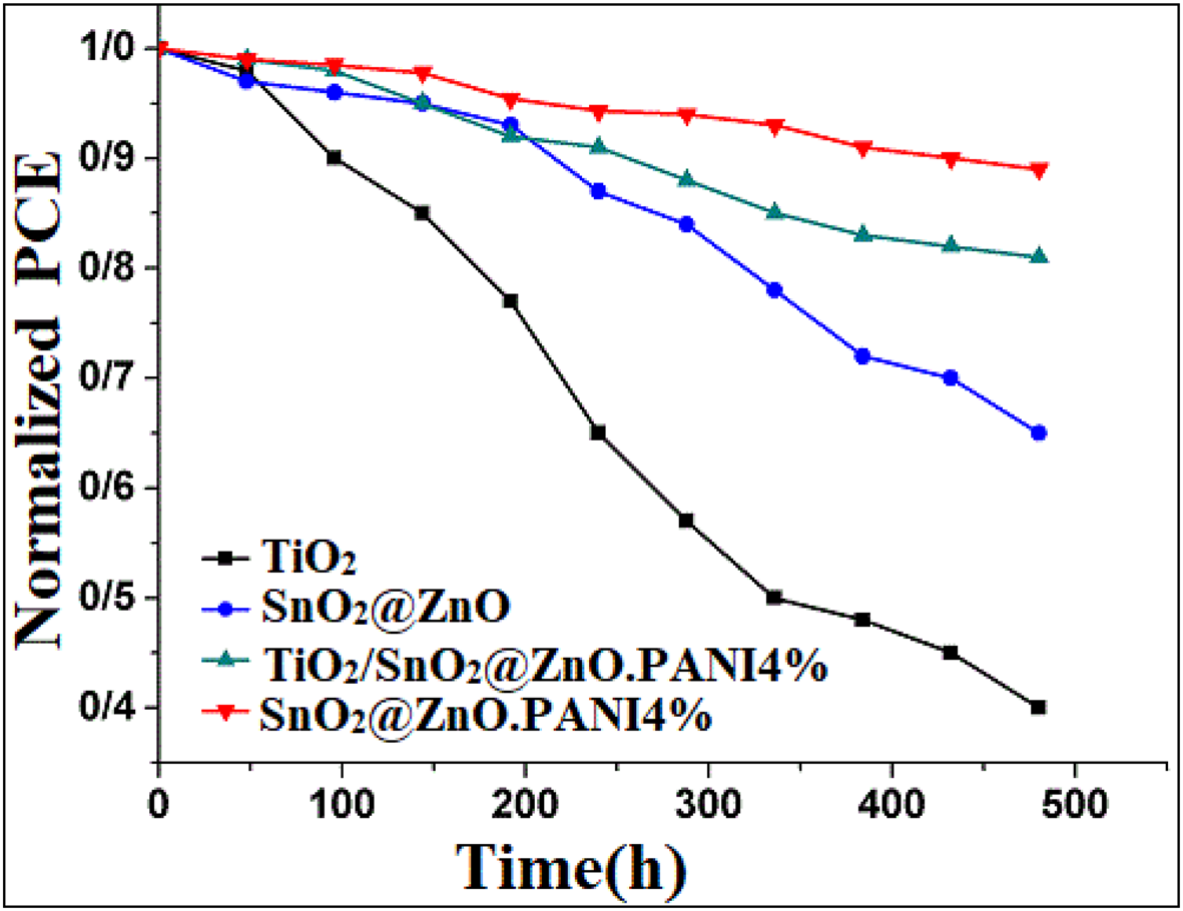
Stability test of perovskite solar cells for 480 h (All devices are placed in ambient air).

Stability is an essential parameter to evaluate the devices performance. Figure 16 illustrates stability tests of the HTL-free PSCs based on TiO_2_, SnO_2_@ZnOnanocomposite, SnO_2_@ZnO.PANI4% nanocomposite, and TiO_2_/SnO_2_@ZnO.PANI4% nanocomposite as ETL with carbon counter electrode under ambient conditions (approximately 25 °C and 45% humidity) for around 480 h. After this time, the PSC with SnO_2_@ZnO.PANI 4% kept 89% of its initial efficiency, whereas the PCE of SnO_2_@ZnO PSC retained only 65% of its original performance. The water molecules lead to the destruction of the MAPbI_3_ layer and cause undesirable morphological changes. Such morphological defects impair the charge transport between the perovskite and the ETM. The presence of PANI in the SnO_2_@ZnO layer helps to better bind to the MAPbI_3_ layer and thus carries extra electrons. As well as improving stability SnO_2_@ZnO.PANI4% -based PSCs are probably due to the size of the enlarged grain with low grain boundaries perovskite, which prevent defects sites that occur mainly at the grain boundaries in light and heat. Moreover, the PSC with TiO_2_/SnO_2_@ZnO.PANI4% kept 81% of its initial efficiency, whereas the PCE of TiO_2_ PSC retained only 40% of its original performance. Therefore, the SnO_2_@ZnO.PANI4% PSC Shows the most stable power output compared with other PSCs with monolayer ETLs.

## Conclusion

In summary, at first, SnO_2_@ZnO nanocomposite was synthesized via a green synthesis way as an electronic transfer layer for the perovskite solar cells. Then, PANI was doped varying amounts in SnO_2_@ZnO nanocomposite as an ETL in a low-temperature method, which inhibited the PbI_2_ crystallization for optimized perovskite films, and improved perovskite stability. PANI additive improves the PCE of PSCs from 11.18 to 14.3%. Furthermore, the optimal PSCs were fabricated by the c-TiO_2_/SnO_2_@ZnO.PANI nanocomposite as ETL with a power conversion efficiency (PCE) of 15.45%. The characterization results show direct evidence that the c-TiO_2_/SnO_2_@ZnO.PANI nanocomposite bilayer ETL improves the transfer properties, electron extraction, and was attributed to the modified interface the band alignment between the perovskite, and ETL layers. Herein, the HTM-free PSCs were utilized based on carbon counter electrodes.

## Data Availability

All data generated or analyzed during this study are included in this published article.
